# Preeclampsia at delivery is associated with lower serum vitamin D and higher antiangiogenic factors: a case control study

**DOI:** 10.1186/s12958-021-00885-z

**Published:** 2022-01-06

**Authors:** David B. Seifer, Geralyn Lambert-Messerlian, Glenn E. Palomaki, Robert M. Silver, Corette Parker, Carol J. Rowland Hogue, Barbara J. Stoll, George R. Saade, Robert L. Goldenberg, Donald J. Dudley, Radek Bukowski, Halit Pinar, Uma M. Reddy

**Affiliations:** 1 Department of Obstetrics and Gynecology, Yale University, New Haven, CT USA; 2 Department of Pathology and Laboratory Medicine, Women and Infants Hospital and the Alpert Medical School at Brown University, Providence, RI USA; 3 Department of Obstetrics and Gynecology, University of Utah Health Sciences, Salt Lake City, UT USA; 4 RTI International, Research Triangle Park, NC USA; 5 Department of Epidemiology, Rollins School of Public Health, Emory University, Atlanta, GA USA; 6 Department of Pediatrics, McGovern Medical School at University of Texas Health Science Center at Houston, Houston, TX USA; 7 Department of Obstetrics and Gynecology, University of Texas Medical Branch at Galveston, Galveston, TX USA; 8 Department of Obstetrics and Gynecology, Columbia University, New York, NY USA; 9 Department of Obstetrics and Gynecology, University of Virginia, Charlottesville, Virginia USA; 10 Department of Women’s Health, Dell Medical School, University of Texas at Austin, Austin, USA

**Keywords:** Preeclampsia, Vitamin D, Endoglin, sFlt-1, Antiangiogenetic factors, Pregnancy

## Abstract

**Background:**

Preeclampsia is characterized by decreased trophoblastic angiogenesis leading to abnormal invasion of spiral arteries, shallow implantation and resulting in compromised placentation with poor uteroplacental perfusion. Vitamin D plays an important role in pregnancy influencing implantation, angiogenesis and placental development. The objective of this study was to determine whether there is an association between serum vitamin D levels, and anti-angiogenic factors at the time of delivery and the occurrence of preeclampsia.

**Methods:**

This nested case control study analyzed frozen serum samples at the time of delivery and related clinical data from women with singleton liveborn pregnancies who had participated in studies of the NICHD Stillbirth Collaborative Research Network. Women with a recorded finding of preeclampsia and who had received magnesium sulfate treatment prior to delivery were considered index cases (*N* = 56). Women without a finding of preeclampsia were controls (*N* = 341).

**Results:**

Women with preeclampsia had 14.5% lower serum vitamin D levels than women in the control group (16.5 ng/ml vs. 19 ng/ml, *p* = 0.014) with 64.5% higher sFlt-1 levels (11,600 pg/ml vs. 7050 pg/ml, *p* < 0.001) and greater than 2 times higher endoglin levels (18.6 ng/ml vs. 8.7 ng/ml, < 0.001). After controlling for gestational age at delivery and maternal BMI, vitamin D levels were 0.88 times lower (*P* = 0.051), while endoglin levels were 2.5 times higher and sFlt-1 levels were 2.1 times higher than in control pregnancies (*P* < 0.001).

**Conclusions:**

Women with preeclampsia at time of delivery have higher maternal antiangiogenetic factors and may have lower maternal serum vitamin D levels. These findings may lead to a better understanding of the underlying etiology of preeclampsia as well as possible modifiable treatment options which could include assuring adequate levels of maternal serum vitamin D prior to pregnancy.

**Supplementary Information:**

The online version contains supplementary material available at 10.1186/s12958-021-00885-z.

## Background

Preeclampsia (PEC) is a major cause of maternal and perinatal morbidity and mortality which is observed in 3–10% of pregnancies. It is characterized by decreased trophoblastic angiogenesis leading to abnormal invasion of spiral arteries, shallow implantation, and compromised placentation with poor uteroplacental perfusion [1]. Women with PEC have been noted to have decreased angiogenic factors and increased antiangiogenic factors compared to women with healthy pregnancies [2, 3].

Vitamin D is a fat-soluble vitamin belonging to the secosteroid family. It is synthesized in the epidermis upon exposure to sunlight and is supplemented by diet. In addition to regulating intestinal calcium and phosphorous absorption and bone metabolism, vitamin D impacts multiple vital organ systems. The female reproductive system has emerged as one such system with both the presence of its receptor, VDR (site of action) and synthesizing enzyme, 1-alpha hydroxylase, in cells of the ovarian granulosa, endometrium, and placenta. Vitamin D plays an important role in pregnancy by influencing implantation, angiogenesis, inflammation, and placental development [4]. Vitamin D alters angiogenic factors (vascular endothelial growth factor or VEGF, placental growth factor or PIGF) and antiangiogenic factors (soluble VEGF receptor-1 or sFlt-1, soluble endoglin sENG) via placental gene expression in preeclampsia [5]. Furthermore, cell culture studies suggest a vital role of vitamin D in the process of angiogenesis [6]. Recent in vitro studies examined the ability of fetal cord blood-derived endothelial progenitor cells to invade established monolayers of human fetal umbilical venous endothelial cells. Fetal cord serum from PEC pregnancies was associated with reduced invasion of endothelial cells into the monolayer as compared to fetal cord serum from uncomplicated pregnancies. This reduction was reversed by addition of vitamin D [7]. Thus, it is hypothesized that low levels of vitamin D may contribute to the decreased trophoblastic angiogenesis seen in preeclampsia.

Twelve observational clinical studies published before 2017 investigated the association between vitamin D deficiency and PEC but yielded inconsistent results. Half found an increased risk of vitamin D deficiency among pregnancies with PEC while others found no association. In the past few years, results of randomized controlled trials have been more consistent [8]. Three such studies noted a positive influence of vitamin D supplementation on the course of preeclampsia while one did not [8]. A 2019 Cochrane review concluded that vitamin D supplementation may reduce the risk of PEC (risk ratio (RR) 0.48, 95% confidence interval (CI) 0.30 to 0.79; 4 trials, 499 women, with moderate-certainty evidence) [9]. A 2020 systematic review and meta-analysis with pooled data of 4777 participants from 27 randomized clinical trials of vitamin D supplementation for prevention of preeclampsia estimated a greater reduced risk of preeclampsia (odds ratio [OR] 0.37, 95% confidence interval [CI]: 0.26, 0.52; I2 = 0%) [10].

A recent randomized control trial [11] designed to determine the effectiveness of a prenatal screening program of Iranian pregnant women in optimizing 25-hydroxyvitamin D (25OHD) levels and preventing pregnancy complications had promising and provocative findings. One group of women underwent vitamin D screening and were provided supplements if their levels were low; the other group was not screened. Among the screened and treated group, 53% of women achieved a 25OHD greater than 20 ng/ml, whereas only 2% of unscreened women had sufficient vitamin D levels. Compared to outcomes in pregnancies with low levels, those with 25OHD greater than 20 ng/ml had a 60% decrease in PEC, 50% decrease in gestational diabetes and 40% decrease in preterm delivery.

A potential oral treatment for vitamin D deficiency is widely available, inexpensive, and safe. For these reasons, it is compelling to examine the relationship between vitamin D status and the pathogenesis of PEC. In the U.S., pregnant women with darker skin on average have lower levels of vitamin D, especially at higher latitudes, caused by melanin in the skin blocking the UVB solar radiation necessary for its synthesis [12]. While differences in vitamin D deficiency related to skin color may help explain the known higher risk of preeclampsia among African American women, these women have seldom been included in vitamin D-birth outcomes research [13]. The majority of studies have been done in Iran, India, Pakistan, and Turkey where skin exposure to sun is limited by cultural norms. Thus, the lack of generalizability of these findings to different geographic locations and cultures supports the need for study in a US based racially heterogeneous sample of pregnant women. The objective of this nested case control study was to determine whether there is an association between serum vitamin D levels and anti-angiogenic factors at the time of delivery and the occurrence of PEC. This dataset was created using frozen serum samples collected at delivery from a US case control study of stillbirths and live births [14].

## Methods

### Original sample/dataset

IRB approval for this study was obtained at participating sites in the parent study and included the use of de-identified pregnancy-related data as well as frozen serum samples obtained from enrolled women at time of delivery. The Yale IRB determined that this was not considered human subjects research since data and samples were deidentified. These study samples and related data were collected by the investigative teams in the NICHD Stillbirth Collaborative Research Network (SCRN). The SCRN attempted to enroll all women who delivered a stillbirth as well as a representative control sample of women with live born infants. Preterm births and infants born to non-Hispanic black women were oversampled due to over-representation in populations of stillbirths. For this reason, a weighting scheme was devised that allowed for the observations in their control group to estimate the general pregnancy population [14].

### Current dataset

The current nested case control study relied only on the SCRN control samples from singleton liveborn pregnancies where sufficient serum was available. Women with chronic hypertension, missing race or missing pre-pregnancy body mass index (BMI) measurements were excluded. Women with a recorded finding of preeclampsia and who received magnesium sulfate treatment prior to delivery were considered index cases. Women without a finding of preeclampsia, regardless of magnesium sulfate treatment status, were considered potential controls. A random sampling of the potential controls was selected based on stratified levels of pre-pregnancy body mass index (< 23, 23–34 and > 34 kg/m2) and gestational age (< 30, 30–36 and > 37 weeks). That is, in each of these nine groups, there were approximately equal ratios of cases to controls. There were 56 cases with preeclampsia.

### Serum sample testing

As previously described, maternal serum was collected at delivery and stored frozen until testing [14]. Total 25-hydroxy vitamin D levels were measured using the automated Liaison platform (DiaSorin, Stillwater, MN) according to manufacturer instructions. The assay sensitivity was 10 ng/mL and inter-assay coefficients of variation were 12.1% at 15 ng/mL, and 7.2% at 49 ng/mL. Soluble endoglin, and soluble flt-1 (also known as soluble vascular endothelial receptor 1 or s-VEGF-R1) were measured using manual enzyme linked immunosorbent assays (ELISA) from R&D Systems (Minneapolis, MN). Lower limits of detection were 0.15 ng/mL and 31 pg/mL, respectively. Samples were run in duplicate. Inter-assay coefficients of variation were tested at low, medium, and high concentrations for each analyte, and all were < 15%. Assays were performed without operator knowledge of which samples were associated with preeclampsia.

### Statistical analyses

Relevant data fields from the original study were identified and extracted. These data were linked to the sample number by study code only. Fields included maternal characteristics such as age, race/ethnicity, pre-pregnancy body mass index, educational level attained, smoking status, and gravidity. Pregnancy outcomes included gestational age at delivery, birthweight, and type of delivery. Other information included the weighting factor and the year of delivery. Weights accounted for oversampling and other aspects of the SCRN study design as well as differential consent based on characteristics recorded on the screened population [14]. Weighted means and standard deviations and proportions were computed for the PEC and control groups and compared using the t-test, for continuous variables and a chi-squared test with continuity correction for categorical variables. Biochemical results were converted to gestational age and maternal BMI adjusted multiples of the median (MoM) to allow for direct comparison. Weighting was not used for converting analyte levels to MoM levels. To examine differences in the MoM levels between cases and controls, a weighted analysis of variance was employed. Correlations were provided with 95% confidence intervals. Statistical significance was at a two-tailed *p*-value of 0.05. Statistical analyses were performed using BMDP (Statistical Solutions, Boston, MA) and graphics produced using GraphPad Prism v8.4.3 (San Diego, CA).

Power analyses were performed using nQuery 8 v8.7.2.0, (Statols, San Diego, CA). Based on previous work [15], the expected vitamin D levels in controls would be about 26 pg/mL, with a logarithmic standard deviation of 0.18. Cases might have levels reduced by about 0.5 SD. Given there were 56 cases available, 74 controls would be needed to find this difference statistically significant at a two-sided *p* = 0.05 level with 80% power. However, this analysis did not include weighting factors which would greatly reduce the power. To account for this, we created a 6:1 match (341 controls to 56 cases).

## Results

Women with preeclampsia were slightly younger (25.8 vs 27.9 years old, *p* = 0.027), more than twice as likely to be primigravida (64% vs. 31%, *p* < 0.001), and had earlier gestational age at delivery (37 vs. 38.4 weeks, *p* = .0015) than women in the control group (Table 1). The case women’s deliveries were more likely to be induced than spontaneous (56% vs. 27%, p < 0.001) and have a lower birth weight (2767 vs. 3291 g) than women in the control group. Pre-pregnancy BMI, level of education, smoking status, gestational diabetes, and racial/ethnicity did not differ significantly between groups. Women with preeclampsia had 14.5% lower serum vitamin D levels than women in the control group (16.5 ng/ml vs. 19 ng/ml, *p* = 0.014) with 64.5% higher sFlt-1 levels (11,600 pg/ml vs. 7050 pg/ml, p < 0.001) and greater than 2 times higher endoglin levels (18.6 ng/ml vs. 8.7 ng/ml, < 0.001).

**Table 1:** Characteristics of the study population and biochemical testing results^a^

Characteristic	All	Preeclampsia	Controls	***p***-value
Number of women	397	56	341	
Weighted number	270	32	238	
Mother’s age (years)^b^	27.7 (5.2)	25.8 (5.1)	27.9 (5.2)	0.027
Pre-pregnancy BMI (kg/m^2^)^b^	28.3 (5.6)	28.6 (6.2)	28.2 (5.9)	0.74
Education (years)^b^	13.5 (2.6)	12.7 (1.9)	13.6 (2.7)	0.055
Non-smoker^c^	88%	87%	90%	0.67
Gestational diabetes	9%	9%	11%	0.79
Prima gravida	35%	64%	31%	< 0.001
GA at delivery (weeks)^b^	38.2 (2.3)	37.0 (2.6)	38.4 (2.3)	0.0015
Birthweight (g)^b^	3228 (576)	2767 (597)	3291 (553)	< 0.001
Race/ethnicity				
White / non-Hispanic	47%	43%	48%	0.71
Black / non-Hispanic	10%	10%	10%	
Hispanic	43%	47%	42%	
Type of Delivery				
Spontaneous	50%	10%	52%	< 0.001
Induced	30%	56%	27%	
No labor	20%	34%	21%	
Endoglin (ng/mL)^d^	9.5 (0.21)	18.6 (0.19)	8.7 (0.19)	< 0.001
sFlt-1 (pg/mL)^d^	7500 (0.33)	11,600 (0.27)	7050 (0.34)	< 0.001
Vitamin D (ng/mL)^d^	19.0 (0.15)	16.5 (0.14)	19.3 (0.15)	0.014

*BMI* body mass index, *GA* gestational age, *sFlt-1* Soluble fms-like tyrosine kinase-1

^a^Using weights to account for over-sampling for preterm birth and non-Hispanic blacks in the original study

^b^Results reported as mean (standard deviation, SD)

^c^Based on cotinine, the major metabolite of nicotine

^d^Geometric mean (logarithmic SD), due to a right skewed distribution

**Fig. 1:**
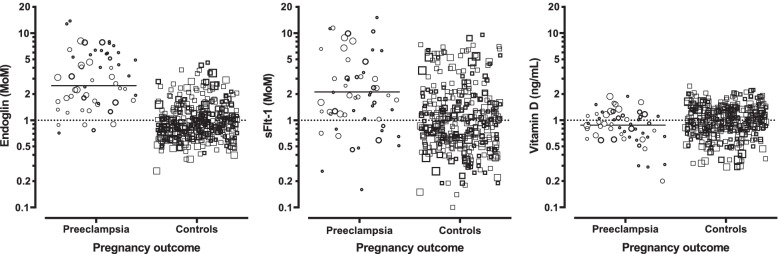
Scatterplot of endoglin, sFlt-1 and vitamin D levels reported as gestational age and body mass index (BMI) adjusted multiples of the median (MoM) in both those pregnancies with preeclampsia and controls (without preeclampsia). The two pregnancy outcome groups are shown on the x-axis with the endoglin, sflt-1 and vitamin D MoM levels on the logarithmic y-axis. Within each group, the data are randomly dithered horizontally to reduce overlap. The size of the symbols indicate the weighting values and have been stratified into five levels. The smallest to largest circles have weights between 0.02 and 0.19, 0.20 to 0.69, 0.70 to 0.89, 0.90 to 0.99 and > 1.00, respectively. The bolding of the symbols indicate the pregnancy delivery prior to 36 weeks gestation. By definition, the controls have a median MoM of 1.00. The weighted median MoM levels for the pregnancies with preeclampsia groups for the three markers are 2.50. 2.11 and 0.88 MoM. The smaller symbols tend to be associated with gestational ages at delivery under 30 weeks

Figure 1 shows gestational age and BMI adjusted analyte levels in both case and control pregnancies. Specifics regarding the gestational age and BMI adjustments can be found in the supplemental materials (sFig. 1, sFigure 2 and sTable 1). Since the results are expressed in MoM levels, the controls are centered at MoM of 1.00. Also, since the vertical logarithmic axis for all three analytes is identical, the spread (standard deviation of the markers in the case and control groups) can be visually compared. Figure 1 also provides insight into the weighting factors. All 397 samples are plotted, with the smaller symbols representing samples with lower weighting factors. In addition, bolded symbols indicate deliveries that occur at less than 37 weeks of gestation. For the endoglin, sFlt-1 and vitamin D MoM levels, the weighted 95th or 5th centiles were > 2.32, > 4.89 and < 0.57 (false positive rates of 5%), respectively. In pregnancies without preeclampsia (controls), the weighted percentages of pregnancies with preeclampsia above these levels were 50, 22 and 6% (detection rates), respectively.

Table 2 shows the difference in the analyte levels in PEC and control pregnancies after accounting for gestational age and maternal BMI (Table 2). The endoglin levels were 2.5 times higher and sFlt-1 levels were 2.1 times higher than in control pregnancies (*P* < 0.001). Adjusted vitamin D levels were 12% lower and of borderline statistical significance (*P* = 0.051). The correlations between the three markers were also examined (Table 3) and both endoglin and sFlt-1 MoM levels had significant correlations in both cases and controls. In contrast, vitamin D levels were not correlated with either endoglin or sFlt-1 in these two groups. Supplemental materials include a scatterplot of all possible combinations of the three markers in cases and controls (sFigure 3).

**Table 2:** Weighted ANOVA results for pregnancies with and without preeclampsia, adjusted for gestational age and maternal body mass index

Marker	Multiple of the median		P
	Preeclampsia	Controls	
Endoglin^a^	2.50 (0.22)	1.00 (0.19)	< 0.001
sFlt-1^a^	2.11 (0.29)	1.00 (0.33)	< 0.001
Vitamin D^a^	0.88 (0.14)	1.00 (0.15)	0.051

^a^adjusted mean, standard deviation

**Table 3:** Correlation coefficients for biochemical markers in pregnancies with preeclampsia (below the diagonal) and in pregnancies without preeclampsia (above the diagonal)

	Endoglin MoM	sFlt-1 MoM	Vitamin D MoM
**Endoglin MoM**		0.495 (*p*<0.001)	-0.024 (*p*=0.69)
**sFlt-1 MoM**	0.455 (*p*<0.0078)		-0.150 (*p*=0.014)
**Vitamin D MoM**	0.064 (*p*=0.72)	0.157 (*p*=0.38)	

## Discussion

This study demonstrates that women with preeclampsia have higher maternal antiangiogenetic factors and may have lower maternal serum vitamin D levels at the time of delivery. The association with lower vitamin D levels is attenuated by and may be related to obesity and gestational age at delivery.

A recent systematic review of observational studies of vitamin D deficiency associated with preterm birth concluded that Vitamin D deficiency in early and late pregnancy may not be associated with preterm birth, while vitamin D deficiency in middle pregnancy is likely to have an important effect on preterm birth [16]. In a nested case control study of preeclampsia in North Carolina, midgestation maternal 25OHD of less than 50 nmol/liter was strongly associated with severe preeclampsia (adjusted odds ratio, 5.41; 95% confidence interval, 2.02–14.52 when compared with midgestation levels of at least 75 nmol/liter) [17].

Further investigation is needed to assess if the relationship between vitamin D and antiangiogenetic factors can be extrapolated to the first trimester of preeclamptic pregnancies during which time abnormal placentation may occur and lead to abnormal or compromised vascular perfusion to the fetus or to the second trimester during which placental growth accelerates. Further investigation could also include angiogenetic factors such as VEGF as well as placenta growth factor (PIGF). If such findings confirm an association between low serum vitamin D levels, higher antiangiogenetic and lower pro-angiogenetic levels this could compel a randomized trial of vitamin D supplementation in vitamin D deficient women during the first two trimesters of pregnancy with the goal of preventing abnormal placentation and supporting placental growth, with the ultimate goal of preventing preeclampsia later in pregnancy.

Limitations of this study include its use of an existing data and frozen serum sample set from a large study designed to examine potential relationship with stillbirth as its primary endpoint. The samples were collected at delivery, limiting the usefulness of the biochemistry results to influence practice. However, it provides a platform to examine the relationships between vitamin D and the angiogenic factors, and our data are hypothesis generating. Confirmation of results using freshly collected samples with preeclampsia as the primary outcome is recommended. Strengths of this study include the wide range of gestational ages at delivery, with a substantial number of observations prior to 30 weeks gestation. Other strengths include rigorous and standardized data collection and a racially, ethnically, and geographically diverse population.

In conclusion, women with preeclampsia at time of delivery were found to have higher maternal antiangiogenetic factors and possibly lower maternal serum vitamin D levels associated with obesity and gestational age at delivery. These findings may lead to a better understanding of the underlying etiology of preeclampsia as well as possible modifiable treatment options which would include assuring adequate levels of maternal serum vitamin D prior to pregnancy.

## Supplementary Information

**Additional file 1: sFigure 1**. Log-linear regression of endoglin, sFlt-1 and vitamin D measurements vs gestational age at delivery. The horizontal axes shows the gestational age at birth in decimal weeks, while the vertical logarithmic axes shows the three analyte levels on a logarithmic scale. Solid line shows the unweighted regression results. Correlations, intercepts, slopes and p-values are contained in sTable 1. **sFigure 2**. Log-linear regression of endoglin, sFlt-1 and vitamin D measurements expressed as multiple of the gestational age-specific medians (MoM) vs the mother’s body mass index. The horizontal axes shows the mother’s body mass index (BMI), while the vertical logarithmic axes shows the three analyte levels on a logarithmic scale. The solid line shows the regression results. The correlations, intercepts, slopes and *p*-values are contained in sTable 1. s**Figure 3**. Scatterplots of Endoglin, sFlt-1 and vitamin D levels at delivery in pregnancies with (left column) and without (right column) preeclampsia, after adjusting for gestational age and body mass index (BMI). **sTable 1**. Parameters for the logarithmic linear regression analyses for the three biochemical measurements to account for the associations with gestational age and body mass index (BMI) among control pregnancies.

## Data Availability

The data that support the findings of this study are available from Dr. David Seifer, but restrictions apply to the availability of these data, which were used under license for the current study, and so are not publicly available. Data are however available from the authors upon reasonable request and with permission of Dr. Uma Reddy and the NICHD Stillbirth Collaborative Research Network.

## Abbreviations

PEC: Preeclampsia
VEGF: Vascular endothelial growth factor
PIGF: Placental growth factor
sFlt-1: Soluble VEGF receptor-1
sENG: Soluble endoglin
SCRN: NICHD Stillbirth Collaborative Research Network
BMI: Body mass index
ELISA: Enzyme linked immunosorbent assay
MoM: Multiples of the median
