# Supplementary material for: Preeclampsia at delivery is associated with lower serum vitamin D and higher antiangiogenic factors: a case control study
Source: Reprod Biol Endocrinol. 2022 Jan 6;20:8. doi: 10.1186/s12958-021-00885-z (PMC8734360; doi:10.1186/s12958-021-00885-z)
Supplement: Supplementary file 1 — Additional file 1: sFigure 1. Log-linear regression of endoglin, sFlt-1 and vitamin D measurements vs gestational age at delivery. The horizontal axes shows the gestational age at birth in decimal weeks, while the vertical logarithmic axes shows the three analyte levels on a logarithmic scale. Solid line shows the unweighted regression results. Correlations, intercepts, slopes and p-values are contained in sTable 1. sFigure 2. Log-linear regression of endoglin, sFlt-1 and vitamin D measurements expressed as multiple of the gestational age-specific medians (MoM) vs the mother’s body mass index. The horizontal axes shows the mother’s body mass index (BMI), while the vertical logarithmic axes shows the three analyte levels on a logarithmic scale. The solid line shows the regression results. The correlations, intercepts, slopes and p-values are contained in sTable 1. sFigure 3. Scatterplots of Endoglin, sFlt-1 and vitamin D levels at delivery in pregnancies with (left column) and without (right column) preeclampsia, after adjusting for gestational age and body mass index (BMI). sTable 1. Parameters for the logarithmic linear regression analyses for the three biochemical measurements to account for the associations with gestational age and body mass index (BMI) among control pregnancies. [file 12958_2021_885_MOESM1_ESM.docx]

**sFigure 1. Log-linear regression of endoglin, sFlt-1 and vitamin D measurements vs gestational age at delivery** The horizontal axes shows the gestational age at birth in decimal weeks, while the vertical logarithmic axes shows the three analyte levels on a logarithmic scale. Solid line shows the unweighted regression results. Correlations, intercepts, slopes and p-values are contained in sTable 1.

**sFigure 2. Log-linear regression of endoglin, sFlt-1 and vitamin D measurements expressed as multiple of the gestational age-specific medians (MoM) vs the mother’s body mass index** The horizontal axes shows the mother’s body mass index (BMI), while the vertical logarithmic axes shows the three analyte levels on a logarithmic scale. The solid line shows the regression results. The correlations, intercepts, slopes and p-values are contained in sTable 1.

s**Figure 3. Scatterplots of Endoglin, sFlt-1 and vitamin D levels at delivery in pregnancies with (left column) and without (right column) preeclampsia, after adjusting for gestational age and body mass index (BMI)**

**sTable 1. Parameters for the logarithmic linear regression analyses for the three biochemical measurements to account for the associations with gestational age and body mass index (BMI) among control pregnancies**

|  | **Logarithmic Linear regression against gestational age** | | | | |
| --- | --- | --- | --- | --- | --- |
|  | **Endoglin**  **(ng/mL)** |  | **sFlt-1**  **(pg/mL)** |  | **Vitamin D**  **(ng/mL)** |
| **Correlation** | 0.449 |  | 0.386 |  | 0.107 |
| **Intercept** | 0.255 |  | 2.834 |  | 1.144 |
| **Slope** | 0.017 |  | 0.0257 |  | 0.0032 |
| **P-value** | <0.01 |  | <0.01 |  | 0.05 |
|  |  |  |  |  |  |
| **Gestational** |  |  |  |  |  |
| **age (weeks)^a^** |  |  |  |  |  |
| 21.0 | 4.09 |  | 2364 |  | 16.3 |
| 42.0 | 9.31 |  | 8192 |  | 19.0 |

|  | **Logarithmic Linear regression against maternal BMI** | | | | |
| --- | --- | --- | --- | --- | --- |
| **Correlation** | 0.12 |  | 0.06 |  | 0.205 |
| **Intercept** | 0.0857 |  | 0.137 |  | 0.147 |
| **Slope** | -0.0037 |  | -0.0034 |  | -0.0055 |
| **P-value** | 0.02 |  | 0.30 |  | <0.01 |
|  |  |  |  |  |  |
| **Body mass index** |  |  |  |  |  |
| **(Kg/m^2^)^b^** |  |  |  |  |  |
| 15.0 | 1.07 |  | 1.22 |  | 1.16 |
| 45.0 | 0.83 |  | 0.96 |  | 0.79 |

^a^ Row entries are the estimated analyte levels at the specified gestational ages based on the regression analyses

^b^ Row entries are the estimated analyte levels at the specified BMI levels based on the regression analyses
